# An Ultrahigh Capacity Graphite/Li_2_S Battery with Holey‐Li_2_S Nanoarchitectures

**DOI:** 10.1002/advs.201800139

**Published:** 2018-05-07

**Authors:** Fangmin Ye, Hyungjun Noh, Hongkyung Lee, Hee‐Tak Kim

**Affiliations:** ^1^ Department of Chemical and Biomolecular Engineering Korea Advanced Institute of Science and Technology (KAIST) 291 Daehak‐ro Yuseong‐gu Daejeon 34141 Republic of Korea; ^2^ Advanced Battery Center KAIST Institute for the NanoCentury Korea Advanced Institute of Science and Technology (KAIST) 335 Gwahangno Yuseong‐gu Daejeon 34141 Republic of Korea; ^3^ Electrochemical Materials & Systems Energy and Environment Directorate Pacific Northwest National Laboratory Richland WA 99352 USA

**Keywords:** concentrated electrolytes, graphite/Li_2_S batteries, holey structures, Li_2_S cathodes, Li_2_S utilization

## Abstract

The pairing of high‐capacity Li_2_S cathode (1166 mAh g^−1^) and lithium‐free anode (LFA) provides an unparalleled potential in developing safe and energy‐dense next‐generation secondary batteries. However, the low utilization of the Li_2_S cathode and the lack of electrolytes compatible to both electrodes are impeding the development. Here, a novel graphite/Li_2_S battery system, which features a self‐assembled, holey‐Li_2_S nanoarchitecture and a stable solid electrolyte interface (SEI) on the graphite electrode, is reported. The holey structure on Li_2_S is beneficial in decomposing Li_2_S at the first charging process due to the enhanced Li ion extraction and transfer from the Li_2_S to the electrolyte. In addition, the concentrated dioxolane (DOL)‐rich electrolyte designed lowers the irreversible capacity loss for SEI formation. By using the combined strategies, the graphite/holey‐Li_2_S battery delivers an ultrahigh discharge capacity of 810 mAh g^−1^ at 0.1 C (based on the mass of Li_2_S) and of 714 mAh g^−1^ at 0.2 C. Moreover, it exhibits a reversible capacity of 300 mAh g^−1^ after a record lifecycle of 600 cycles at 1 C. These results suggest the great potential of the designed LFA/holey‐Li_2_S batteries for practical use.

Because of its high theoretical specific capacity (1166 mAh g^−1^), fully lithiated Li_2_S possesses a high potential to replace low‐capacity transition metal oxides as a cathode active material for next generation lithium batteries. Compared with sulfur‐based^1^, ^2^, ^3^, ^4^, ^5^, ^6^, ^7^, ^8^, ^9^, ^10^ and Li_2_S*_x_*‐based (*x* > 2)^11^, ^12^, ^13^ cathodes, Li_2_S cathodes have a unique merit of being paired with lithium‐free anode (LFA), e.g., graphite,^14^, ^15^, ^16^, ^17^, ^18^ tin (Sn),^19^ silicon (Si),^20^, ^21^, ^22^, ^23^, ^24^ and metal oxides,^25^, ^26^ which are more stable and safer than Li metal electrodes. Moreover, Li_2_S‐based electrodes are advantageous in maintaining their structural integrity because the as‐prepared Li_2_S electrodes are at their maximal volume. As an LFA for Li_2_S‐based batteries, graphite can provide a higher cycling stability than conversion‐type Sn and Si anodes due to its smaller volume expansion (9–13%) upon lithiation. These incentives have motivated the development of graphite/Li_2_S batteries in recent years.^14^, ^15^, ^16^, ^17^, ^18^ However, two intractable barriers are impeding the progress. These are 1) the high potential barrier against Li_2_S oxidation during the first charge step and 2) the large irreversible capacity caused by the formation of SEI on the graphite surfaces.^15^, ^18^ These barriers are responsible for the low specific capacities of graphite/Li_2_S batteries.

Because of its low electronic and ionic conductivity, bulk Li_2_S powder shows a high potential barrier when activating Li_2_S cathode‐based batteries. The high potential barrier represents the difficulty in extracting lithium ion from Li_2_S,^27^ which limits the depth of charging, thereby causing the low Li_2_S utilization. To address this issue, the size reduction of Li_2_S powders^27^, ^28^, ^29^, ^30^, ^31^, ^32^ and the fabrication of various Li_2_S composites^33^, ^34^, ^35^, ^36^, ^37^, ^38^, ^39^, ^40^ from Li_2_S powder have been widely reported. However, the use of commercial Li_2_S powder does not reduce the cost of Li_2_S cathodes for consumer‐oriented batteries. The low‐cost production is highly important for energy storage systems and electric vehicle applications where battery cost reduction is a key driver for their successful implementation. As a cost‐effective route, the fabrication of Li_2_S cathodes by a carbothermal conversion of Li_2_SO_4_ has been reported recently,^41^, ^42^, ^43^, ^44^, ^45^, ^46^, ^47^, ^48^, ^49^ which also provides a chance to scale the fabrication of Li_2_S electrodes. However, due to the high conversion temperature required, most of the Li_2_SO_4_‐converted Li_2_S cathodes have a high potential barrier upon their activation. Therefore, to achieve a high Li_2_S utilization of Li_2_S cathodes with an intrinsically high potential barrier is a great challenge to advance the graphite/Li_2_S batteries.

On the other hand, electrolyte design is another critical issue for achieving high‐capacity graphite/Li_2_S batteries. Previously, the electrolytes containing 1 m bistrifluoromethanesulfonimide lithium salt (LiTFSI) in dioxolane (DOL)/dimethoxymethane (DME) and LiNO_3_ additives were reported for graphite/Li_2_S batteries.^18^ However, such ether‐based electrolytes are known to decompose above 3.5 V,^50^ which is unsuitable to Li_2_S cathodes with a high potential barrier. As a means to improve the oxidative stability of the ether‐based electrolyte, a highly concentrated electrolyte (5 m LiTFSI in DME) was suggested for lithiated graphite/sulfur batteries.^51^ The use of highly concentrated electrolyte is also beneficial in reducing the polysulfide shuttle^52^, ^53^, ^54^, ^55^ and the irreversible lithium loss for SEI formation on the graphite surface,^51^, ^56^, ^57^, ^58^ both of which can enhance the Coulombic efficiency (CE) of the battery. In spite of these advantages, the highly concentrated electrolytes usually have high viscosity and low ion conductivity,^58^, ^59^ which can result in the poor electrolyte wetting of porous electrodes and low‐rate capability, respectively. Therefore, the careful tuning of lithium salt concentration and solvent composition is needed.

Against the backdrop, we report a novel holey‐Li_2_S nanoarchitecture fabricated by a facile, low‐cost, and solid‐state carbothermal reaction of Li_2_SO_4_, and a high‐performance graphite/Li_2_S battery with the holey‐Li_2_S‐based cathode as well as a conventional graphite electrode, and a concentrated DOL‐rich electrolyte. The unique holey nanostructure, which can expand the Li_2_S/electrolyte interface, facilitates the oxidation of Li_2_S during the initial activation process. A 3 m LiTFSI DOL‐rich electrolyte was rationally designed that considered the balance among ionic conductivity, oxidation stability, and SEI formation on the graphite anode. In addition, due to the use of the graphite anode instead of the Li metal anode, problematic polysulfide shuttle can be eliminated accordingly. The combined approach results in a graphite/holey‐Li_2_S battery that has an ultrahigh initial discharge capacity of 810 mAh g^−1^ at 0.1 C and the long lifecycle over 600 cycles at a 1 C rate. These performances are far superior to those of the previous studies on graphite/Li_2_S batteries (Table S1, Supporting Information) and even better than conventional lithium ion batteries in terms of specific energy (Table S2, Supporting Information). This suggests that the graphite/Li_2_S battery with holey‐Li_2_S nanoarchitectures and concentrated DOL‐rich electrolyte is highly promising for practical applications.

The novel Li_2_S cathode consisting of micrometer‐sized Li_2_S particles with a hole (holey‐Li_2_S) and carbon nanotube (CNT) network is fabricated from a low‐cost commercial Li_2_SO_4_·H_2_O via a facile two‐step method (see the Experimental Section for the detailed fabrication process). First, plate‐shaped Li_2_SO_4_ particles embedded in a CNT network (plate‐Li_2_SO_4_/CNT) (**Figure** **1**, left) are obtained by the precipitation of the Li_2_SO_4_·H_2_O aqueous solution in CNT containing ethanol solution and subsequent filtration. Second, the as‐prepared plate‐Li_2_SO_4_/CNT electrodes are converted to holey‐Li_2_S/CNT electrodes (Figure 1, middle) via a carbothermal reduction reaction under N_2_ gas at 700 °C for 3 h. The resulting holey‐Li_2_S/CNT electrode is freestanding, and thus, can be used as a cathode without an additional binder. The pristine holey‐Li_2_S particles embedded in the CNT network are oxidized to higher‐order sulfur species, and these are redistributed in the CNT network during the initial charge process (Figure 1, right). More interestingly, with the conversion of these holey‐Li_2_S particles, micrometer‐sized pores can be formed accordingly in the electrodes, which, in the subsequent discharge/charge process, could enhance the lithium ion transport and improve the rate capability.

**Figure 1:**
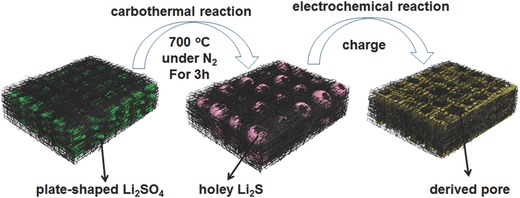
Schematic illustration of the structural changes upon carbothermal conversion from Li_2_SO_4_ to Li_2_S and upon the initial charge process from Li_2_S to sulfur.

**Figure** **2** shows the microstructures and morphologies of the as‐prepared Li_2_SO_4_/CNT (Figure 2a–d) and the as‐converted holey‐Li_2_S/CNT electrode (Figure 2e,f). The scanning electron microscope (SEM) image of the as‐prepared Li_2_SO_4_/CNT electrode (Figure 2a) shows that the plate‐shaped Li_2_SO_4_ precipitates are uniformly distributed in the CNT network. The Li_2_S plates are quite uniform in size and shape, which is contrasted by the pristine Li_2_SO_4_·H_2_O particles (Figure S1, Supporting Information). The X‐ray diffraction (XRD) pattern for the as‐prepared Li_2_SO_4_/CNT electrode (Figure S2, Supporting Information) indicates that the plate‐shaped architectures are a mixture of Li_2_SO_4_·H_2_O (Joint Committee on Powder Diffraction Standards (JCPDS) card No. 15–0873) and Li_2_SO_4_ (JCPDS card No. 20–0640). A magnified SEM image (Figure 2b) reveals that the plate‐Li_2_SO_4_ has a rectangular shape with a dimension of around 1.5 × 1 × 0.3 µm. As shown in Figure 2b, the CNTs and the plate‐Li_2_SO_4_ are in keen contact, which could facilitate the carbothermal conversion of the plate‐Li_2_SO_4_ into Li_2_S. The transmission electron microscope (TEM) images (Figure 2c,d) further confirm the structural feature of the Li_2_SO_4_ precipitate. The formation of such a plate structure can possibly profit from the use of negatively charged poly(acrylic) acid (PAA) as a surfactant, which has a strong affinity with Li ion and thus favors the formation of Li_2_SO_4_ plates. The use of neutral poly(vinyl pyrrolidone) instead of PAA led to the formation of few micrometer‐long strip‐shaped Li_2_SO_4_ (Figure S3, Supporting Information).^60^

**Figure 2:**
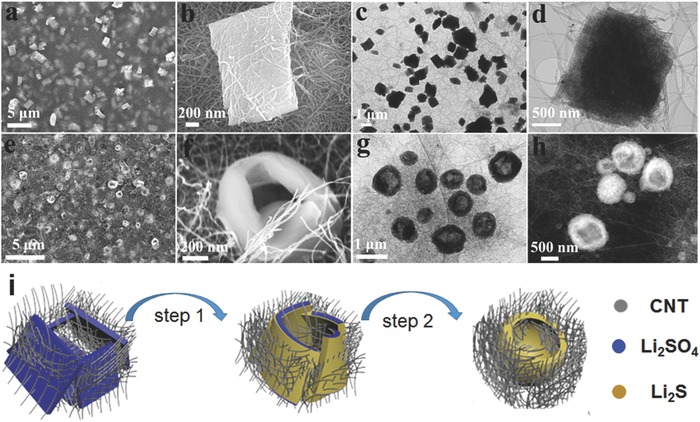
Structural characterization of Li_2_SO_4_/CNT and Li_2_S/CNT electrodes: a,b) SEM images and c,d) TEM images of the as‐prepared Li_2_SO_4_/CNT electrode. e,f) SEM images, g) TEM image, and h) STEM image of the as‐prepared Li_2_S/CNT electrode. i) A suggested mechanism for the formation of the holey‐Li_2_S nanoarchitecture.

Complete conversion from Li_2_SO_4_ to Li_2_S during the carbothermal conversion is confirmed by the XRD pattern of the as‐converted Li_2_S electrode (Figure S4, Supporting Information), which shows that the diffraction peaks perfectly match with those of the cubic Li_2_S phase (JCPDS card No. 26–1188). Interestingly, as shown in the SEM image of the as‐converted Li_2_S electrode (Figure 2e), the plate‐shaped Li_2_SO_4_ particle was transformed to a doughnut‐shaped Li_2_S particle with a hole (holey‐Li_2_S) during the carbothermal reduction process. This is different in shape from the shapes of the previously reported Li_2_S particles derived from Li_2_SO_4_ compounds.^51^, ^52^, ^53^, ^54^, ^55^, ^56^, ^57^, ^58^, ^59^ A magnified SEM image (Figure 2f) reveals that the holey‐Li_2_S nanoarchitecture has a wall thickness of 100–150 nm and a hole diameter of 200–500 nm. Evolution of the holey structure was further evidenced by the TEM image (Figure 2g) and scanning transmission electron microscopy (STEM) image (Figure 2f). It should be noted that the holey structure is beneficial in improving the lithium ion transfer between the electrolyte and Li_2_S particle due to the expanded interface.

The inscrutable structural change from plate to doughnut can be understood as a self‐assembly of the plates with the consumption of the near‐by CNT matrix (Figure 2i). As indicated in the carbothermal reduction reaction equation,^43^ Li_2_SO_4_ + 2 C →  Li_2_S + 2 CO_2_, the CNTs adjacent to the Li_2_SO_4_ plates (left) are first consumed and the skin of the Li_2_SO_4_ plates is converted to Li_2_S, preventing the direct contact between the surrounding CNTs and inner Li_2_SO_4_. However, the reaction between the generated CO_2_ and CNT (CO_2_ +  2 C →  2 CO) produces CO,^61^ and the carbothermal reduction of the inner Li_2_SO can occur due to the strong reducing power of CO (Li_2_SO_4_ + 4 CO →  Li_2_S + 4 CO_2_). The carbothermal conversion accompanies the compaction of the plates, and due to the absence of the nearby CNTs, the resulting Li_2_S plates become closer and eventually merge into a holey structure (right). The absence of CNT inside the hole suggests that the removal of CNT drives the assembly of the Li_2_S plates. The jointing of two plates observed for the holey‐Li_2_S particle (Figure S5, Supporting Information) further supports the self‐assembled process of Li_2_S plates.

For comparison, a Li_2_S particulate without any holes was prepared by further heating the above holey‐Li_2_S nanoarchitectures at 1000 °C for 3 h. Since the applied temperature is higher than the melting point of Li_2_S (938 °C), the holey structure was disrupted and a nonholey Li_2_S particulate was obtained, as shown in the SEM image and XRD pattern of the nonholey Li_2_S (solid‐Li_2_S) (Figures S6 and S7, Supporting Information).

To achieve high performance graphite/Li_2_S batteries, the selection of liquid electrolytes presents a challenge. In this work, we paid attention to 3 m LiTFSI DOL/DME electrolytes by considering the balance between high oxidation stability, high ionic conductivity, and the compatibility with Li_2_S cathode and graphite anode. As shown in **Figure** **3**a, the 3 m LiTFSI DOL/DME electrolytes with different DOL/DME volume ratios (DOL/DME = 100/0, 85/15, 75/25, 50/50, and 0/100) showed higher oxidation stabilities compared with 1 m LiTFSI in DOL/DME = 50/50 with 0.2 m LiNO_3_, which is conventionally used for Li_2_S or sulfur batteries. Although the oxidative stability might be further improved by increasing the LiTFSI concentration, there is a significant loss in ionic conductivity as previously reported.^59^ and due to a solubility limit of LiTFSI salt at room temperature, the concentrations over 3 m could not be achieved (Figure S8, Supporting Information).

**Figure 3:**
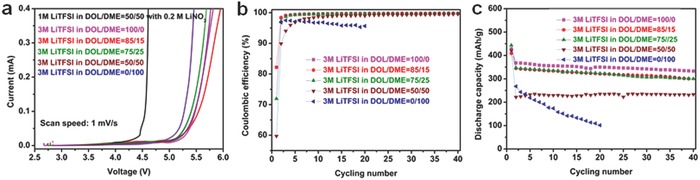
Electrolyte characterizations. a) Oxidation stability of the 3 m LiTFSI‐DOL/DME electrolytes with various DOL/DME ratios (100/0, 85/15, 75/25, 50/50, and 0/100 in volume) and conventional electrolytes (1 m LiTFSI in DOL/DME = 50/50 with 0.2 m LiNO_3_). b) The CEs and c) cycling stabilities of the graphite/Li batteries with the 3 m LiTFSI DOL/DME electrolytes with various DOL/DME ratios.

In order to check the compatibility of the 3 m TFSI DOL/DME electrolytes with graphite anode, the CEs during the 0.1 C rate cycling were measured for the graphite/Li batteries with the 3 m LiTFSI DOL/DME electrolytes. As shown in Figure 3b, the DOL/DME = 100/0, 85/15, and 0/100 electrolyte have an initial CE of 82.2%, 82.2%, and 82.6%, respectively. These values are much higher than those of the DOL/DME = 75/25 (71.9%) and 50/50 (≈59.7%) and the electrolytes with a concentration of 1 m (16.3%) and 2 m (65.4%) LiTFSI in DOL/DME = 85/15, respectively (Figure S9, Supporting Information), suggesting a relatively smaller irreversible lithium consumption for SEI formation on graphite for the three electrolytes. For the DOL‐rich electrolytes (DOL/DME = 100/0, 85/15, and 75/25), the CEs reached over 99% after a few cycles, indicating the formed SEI films are quite stable. For the DOL/DME = 50/50 electrolytes, CEs gradually increased with the cycle, suggesting a gradual coverage of the SEI film on the graphite anode with the cycle. The DOL/DME = 0/100 electrolyte exhibited a fast CE fade with the cycle, which indicates that the SEI layer formed by 3 m TFSI in DME is not dense enough to prevent the cointercalation of the lithium ion and DME molecular into graphite.^62^ The low irreversible capacity loss for the DOL‐rich electrolytes can be associated with the formation of thin and uniform polymeric layer on graphite in DOL‐based electrolytes. The advantage of the DOL‐rich electrolytes is also supported by the good cycling stabilities for the various electrolytes (Figure 3c). For the DOL‐rich electrolytes (DOL/DME = 100/0, 85/15, and 75/25), highly stable cycling performances with discharge capacities higher than 350 mA g^−1^ were obtained over 40 cycles, which could be ascribed to a compact and uniform SEI layer derived from the DOL solvent^63^ and an electrochemically stable SEI layer.^64^ On the other hand, the DOL/DME = 0/100 electrolyte showed a fast capacity fade (Figure 3c, blue) due to the cointercalation. A more stable cycling performance was observed for the DOL/DME = 50/50 electrolyte, meaning that the introduction of DOL solvent improves the SEI layer. However, the discharge capacities were quite low (≈220 mAh g^−1^), which can be attributed to the large irreversible capacity at the first charging step. Therefore, the DOL‐rich electrolytes (DOL/DME = 100/0, 85/15, and 75/25) are more suitable for the graphite anode.

The compatibilities with Li_2_S cathode for the three DOL‐rich electrolytes were assessed with Li/holey‐Li_2_S batteries. As shown in Figure S10 in the Supporting Information, the first discharge capacity was 773, 880, 792, and 870 mAh g^−1^ for DOL/DME = 100/0, 85/15, and 75/25 and 50/50, respectively. The discharge voltage plateau was 2.05, 2.10, 2.10, and 2.10 V for DOL/DME = 100/0, 85/15, 75/25, and 50/50 respectively. However, the initial potential barrier of Li_2_S cathode increases with the increase of DME content in the electrolyte and, when the content of DME arrives at 100%, the initial charge/discharge process quickly completed with a very low charge/discharge capacity (Figure S10e, Supporting Information). These results indicate that a certain amount of DME is needed to attain the compatibility with the Li_2_S cathode, as previously observed for Li/sulfur batteries.^65^, ^66^ As compared (Table S1, Supporting Information), the ionic conductivity was the highest for DOL/DME = 85/15 among the three 3 m electrolytes and the lower concentrated electrolytes. Therefore, taking the above results into consideration, the 3 m LiTFSI DOL/DME = 85/15 electrolyte was selected for the graphite/holey‐Li_2_S battery.

**Figure** **4**a compares the initial charge/discharge curves at the 0.1 C rate for the graphite/holey‐Li_2_S and graphite/solid‐Li_2_S batteries. The holey‐Li_2_S cathode showed a lower initial potential barrier than the solid‐Li_2_S electrode, which means that the holey structure facilitates the lithium extraction from Li_2_S. The charging capacity for the first charging with a cut‐off voltage of 3.8 V was 1166 mAh g^−1^ for the graphite/holey‐Li_2_S and 750 mAh g^−1^ for the graphite/solid‐Li_2_S. It clearly shows that, by introducing the submicrometer scale hole to the Li_2_S particle, the charging overpotential for the Li_2_S oxidation can be considerably lowered and a higher depth of charging can be obtained.

**Figure 4:**
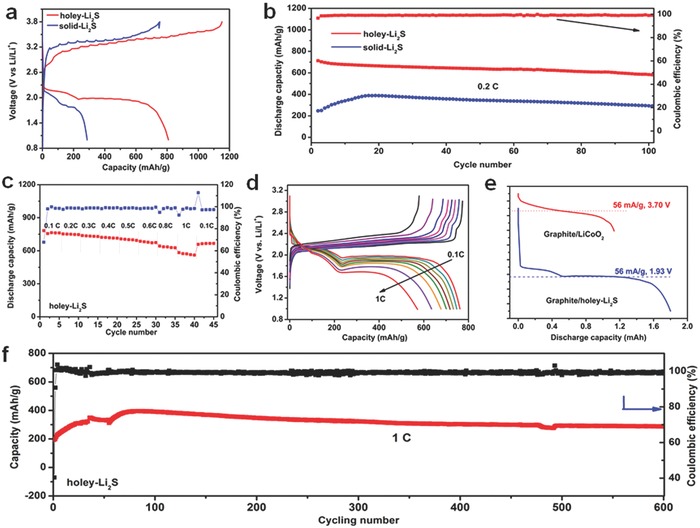
a) The initial charge/discharge curves at 0.1 C and, b) cycling performance at a 0.2 C for the graphite/holey‐Li_2_S and graphite/solid Li_2_S batteries. c) Rate capability and d) the charge/discharge curves at various C rates for the graphite/holey‐Li_2_S battery. e) Comparison of the energy densities between the graphite/holey‐Li_2_S battery and graphite/LiCoO_2_ battery. f) Extended cycling test at 1 C for the graphite/holey‐Li_2_S battery.

The cycling performances of the graphite/holey‐Li_2_S battery and graphite/solid‐Li_2_S battery at 0.2 C after the initial activation were investigated (Figure 4b). The graphite/holey‐Li_2_S battery delivered a discharge specific capacity of 712 mAh g^−1^ at the first cycle and 583 mAh g^−1^ at 100 cycles, which is the highest value among the graphite/Li_2_S batteries ever reported (Table S2, Supporting Information). The graphite/holey‐Li_2_S battery showed a lower capacity fade rate (0.184%/cycle) than the Li metal/holey‐Li_2_S battery (0.414%/cycle) (Figure S11, Supporting Information), demonstrating the benefit of using graphite instead of Li metal in terms of cycling stability. The CEs of the graphite/holey‐Li_2_S battery were maintained above 99% during the whole cycles, which is contrasted by the CEs around 98% for the Li /holey‐Li_2_S battery (Figure S8, Supporting Information). It indicates that the polysulfide shuttle can be suppressed in the graphite/holely‐Li_2_S battery. To further clarify the polysulfide shuttle issue, the lithium metal and graphite electrode of the Li_2_S batteries were analyzed by X‐ray photoelectron spectroscopy (XPS) after the initial charge. As shown (Figure S12, Supporting Information), the peaks from the insoluble Li_2_S/Li_2_S_2_ were clearly detected for the lithium metal surface electrode, while these peaks were unseen for the graphite surface, ensuring the prevention of polysulfide shuttle in the graphite/Li_2_S battery. The discharge capacity of the graphite/solid‐Li_2_S battery gradually increased from 237 to 450 mAh g^−1^ during the first 30 cycles, followed by a mild capacity fade. The initial capacity increase in the early cycles suggests that the unactivated Li_2_S particles gradually decomposed during the cycle. In spite of the additional Li_2_S activation, the maximum discharge capacity was still far lower than that of the holey‐Li_2_S electrode.

The rate capability of the holey‐Li_2_S electrode was evaluated by investigating the discharge capacities for five cycles at a discharge rate with a successively increasing discharge rate as 0.1, 0.2, 0.3, 0.4 0.5, 0.6, 0.8, and 1 C and returning to 0.1 C. As shown in Figure 4d, the averaged discharge capacity was 760, 749, 734, 717, 700, 677, 632, and 570 mAh g^−1^ for the discharge rate of 0.1, 0.2, 0.3, 0.4 0.5, 0.6, 0.8, and 1 C, respectively. When the discharge current density returned to 0.1 C, a high discharge capacity of 664 mAh g^−1^ was recovered. The capacity retention from the C rate increase from 0.1 to 1 C was 75%. In addition, the graphite/holey‐Li_2_S battery showed a high discharge potential plateau of 1.99 V at 0.1 C and 1.68 V at 1 C (Figure 4e), indicating a high power and energy density for the holey‐Li_2_S/CNT electrode. The exceptionally excellent rate capacity can be attributed to the multiscale porosity of the activated holey‐Li_2_S electrode. After the initial activation, micrometer‐sized pores were generated with the decomposition of the holey‐Li_2_S particles (Figure S13, Supporting Information), which will be described in a later section. For sulfur cathodes, the efficacy of the multiscale porosity with micrometer and submicrometer pores in enhancing the rate capability was previously demonstrated.^67^

The superiority of the graphite/holey‐Li_2_S battery can be further supported by a comparison of battery energy densities between the graphite/LiCoO_2_ and graphite/holey‐Li_2_S batteries (Figure 4e; Table S3, Supporting Information). The areal capacities of the two cathodes were controlled to be identical for fair comparison. As marked in Figure 4e, the energy density (based on the total mass of cathode and anode) at a current density of 56 mA g^−1^ (based on whole cathode mass) is 270 Wh kg^−1^ for the graphite/holey‐Li_2_S battery and 206 Wh kg^−1^ for the graphite/LiCoO_2_ battery, respectively. The comparison indicates that the holey‐Li_2_S cathodes can exceed conventional metal oxide cathode in terms of energy density.

Figure 4f shows an extended cycling stability test at 1 C for the graphite/holey‐Li_2_S battery. When the current density was increased to 1 C after the initial activation at 0.1 C for the cycling, the discharge capacity at the first cycle was as low as 195 mAh g^−1^. This is because the redistribution of the sulfur species over the CNT network was not fully proceeded during the initial activation. However, the discharge capacity gradually increased up to a maximum value of 400 mAh g^−1^ after 70 cycles, which was probably due to a gradual redistribution. To our interest, the discharge capacity of 300 mAh g^−1^ was maintained at 600 cycles with a high CE of 99%, which firmly demonstrates the merit of the graphite/holey‐Li_2_S battery in terms of discharge capacity and cycling stability.

According to the initial electrochemical reaction of Li_2_S cathode:Li_2_S → S + 2Li^+^ + 2e^−^, the original Li_2_S is converted into lithium polysulfides and sulfur upon the initial activation process. Accordingly, the electric energy is stored in the battery system, which can be used in the discharge process. Therefore, the initial activation process is highly critical to the electrochemical performances of Li_2_S batteries. To further understand the structural and electrochemical changes during the initial activation for the holey‐Li_2_S and solid‐Li_2_S cathodes, SEM, XRD, and electrochemical impedance spectroscopy (EIS) analysis were conducted for the two electrodes, and the results are compared in **Figure** **5**. After the initial activation, the holey‐Li_2_S particles completely disappeared (Figure 5a); however, some portion of the solid‐Li_2_S particles remained in the CNT matrix as shown in the SEM images taken after the initial activation (Figure 5b). The XRD pattern of the holey‐Li_2_S electrode after the initial activation did not show any peaks from crystalline Li_2_S (Figure 5c), indicating that the crystalline Li_2_S is completely decomposed (more easily decomposed for amorphous Li_2_S during charging^68^) and the charged sulfur products are in their amorphous state. However, the XRD patterns from Li_2_S were clearly seen (Figure 5d) after the initial activation process for the solid‐Li_2_S electrode, indicating the presence of undecomposed, residual Li_2_S. As shown in Figure 5e, the impedances of the two electrodes were nearly identical before the initial activation. However, after the initial activation, the impedances of the two batteries became very different (Figure 5f). For the holey‐Li_2_S cathode, the semicircles were significantly reduced, indicating a faster charge transfer reaction after the activation. In contrast, for the solid Li_2_S cathode, two large semicircles and a long low frequency tail appeared after the activation. The small semicircle for the activated holey‐Li_2_S cathode is in good agreement with the formation of amorphous charged sulfur species and effective redistribution of these species over the CNT matrix with the multiscale porosity. The appearance of the two semicircles may reflect the existence of decomposed and undecomposed regions in the CNT matrix. The EIS results indicate that the holey‐Li_2_S structure is quite effective in achieving a high Li_2_S utilization and constructing high‐performance LFA/Li_2_S batteries.

**Figure 5:**
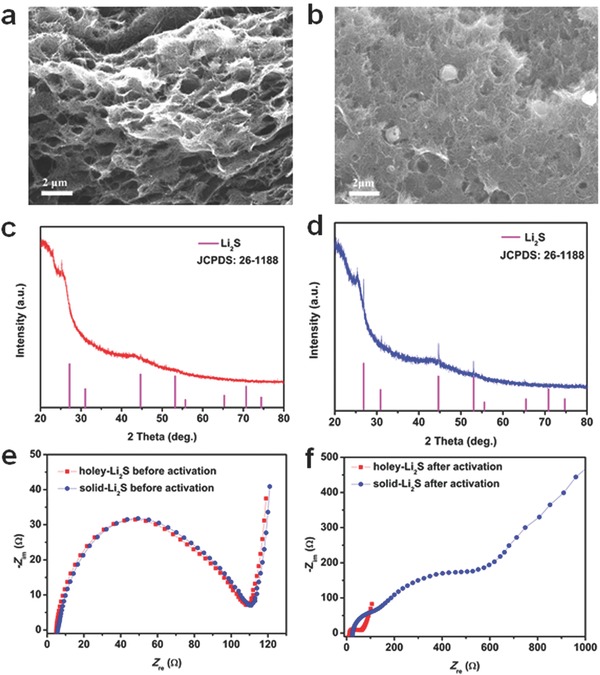
Characterization of the holey‐Li_2_S and solid‐Li_2_S cathodes after initial charge process. a) SEM image and b) XRD pattern for the holey‐Li_2_S cathode. c) SEM image and d) XRD pattern for the solid‐Li_2_S electrode. Comparison of the Nyquist plots of the AC impedances for the holey‐Li_2_S and solid‐Li_2_S electrodes e) before and f) after the initial activation.

In summary, a novel holey‐Li_2_S nanoarchitecture was achieved by the newly invented carbothermal conversion process, which features the formation of plate‐shaped Li_2_SO_4_ particles and the self‐assembly of the plates during the carbon thermal conversion from Li_2_SO_4_ to Li_2_S. In order to reduce the electrolyte decomposition in the Li_2_S cathode at high potentials and the irreversible capacity loss of graphite anode, 3 m LiTFSI in DOL‐rich DOL/DME electrolytes were newly designed. With the combination of the holey‐Li_2_S cathode and the concentrated, DOL‐rich electrolyte, the resulting graphite/holey‐Li_2_S battery provided a record high specific capacity of 810 mAh g^−1^ at 0.1 C and exhibited excellent cycling stability over 600 cycles at 1 C rate. The systematic variations of the Li_2_S structure, electrolyte composition, and anode material (graphite and Li metal) indicate that the high performances of the graphite/holey‐Li_2_S battery can be attributed to the three cooperative contributions; 1) The holey‐Li_2_S nanostructure which facilitates the decomposition of Li_2_S particles during the initial activation, 2) the formation of a stable SEI layer on graphite with the electrolyte, and 3) the prevention of the polysulfide shuttle due to the use of the graphite anode. We believe that the novel holey‐Li_2_S nanoarchitectures and the electrolyte design can boost the development of high‐energy LFA/Li_2_S batteries for practical applications.

## Experimental Section

*Fabrication of Electrode Materials*: In this work, the commercial lithium sulfate monohydrate (Li_2_SO_4_·H_2_O, Sigma‐Aldrich, >99%), PAA (Sigma‐Aldrich), ethanol (EMD millipore corporation), and poly(vinyl pyrrolidone) (PVP, *M*_w_ = 40 000, Sigma‐Aldrich) were used without further purification.

In a typical experiment, the freestanding holey‐Li_2_S/CNT electrodes were fabricated by the following two steps. First, the sandwich‐typed plate‐Li_2_SO_4_/CNT electrodes were prepared by a precipitation method. Detailedly, an 80 mg of Li_2_SO_4_·H_2_O powder was dissolved into a 5 mL of deionized water upon stirring to form a transparent Li_2_SO_4_ solution. At the same time, a 25 mg of CNT and a 100 mg of PAA were in turn added to a 50 mL of absolute ethanol to form a uniformly dispersed CNT suspension via a sonication of 30 min. Then, another CNT suspension was prepared by the same treatment (Recipe: 10 mg CNT, 20 mg PVP, and 20 mL absolute ethanol). After that, the suspension containing a 25 mg of CNT mixed with a more 50 mL of ethanol (total volume: 100 mL) and the prepared Li_2_SO_4_ aqueous solution were soaked in an iced water bath for 30 min upon stirring. Followed this step, the icy Li_2_SO_4_ solution was transferred to a syringe and was injected slowly to the icy CNT suspension upon stirring to obtain a uniform Li_2_SO_4_/CNT suspension. Finally, the Li_2_SO_4_/CNT suspension was used to fabricate a sandwich‐typed Li_2_SO_4_/CNT film by a vacuum filtration (The unused CNT suspension was evenly divided into two parts and was filtrated to as a bottom and an upper CNT layer, respectively. The as‐prepared Li_2_SO_4_/CNT suspension was filtrated into the two CNT layers). The as‐fabricated sandwich‐typed film was peeled off and dried, and was punched into disks with a diameter of 12 mm for further drying at room temperature for overnight. Second, the holey‐Li_2_S/CNT electrodes were obtained by a carbothermal reaction. Operationally, the as‐prepared Li_2_SO_4_/CNT electrodes were put into the tube furnace under a flowing N_2_ at 700 °C for 3 h and were converted into final holey‐Li_2_S/CNT electrodes. The solid‐Li_2_S/CNT electrodes were obtained via a further heat treatment of holey‐Li_2_S/CNT at 1000 °C for 3 h. The Li_2_S content and Li_2_S area loading in the two electrodes according to the mass change of before and after dissolution of Li_2_S into ethanol and deionized water are around 48 wt% and 2.0–2.25 mg cm^−2^, respectively.

*Microstructure Characterization*: The crystalline phase structures of all the converted electrodes were characterized by XRD (Smart lab). The morphology and structure of the electrodes were characterized by SEM (S4800) and TEM (Tecnai F30 ST). XPS characterization was carried out on an X‐ray photoelectron spectroscopy (Kα). EIS data were collected in a frequency range of 1 MHz to 10 Hz using an alternating current (AC) impedance analyzer with amplitude of 10 mV. The electrochemical measurements were carried out on a battery cycler (TOSCAT‐3000U) at 25 °C.

*Electrochemical Characterization*: Electrochemical performances of the electrodes were evaluated by using the assembled button‐type batteries. The holey‐Li_2_S/CNT and solid‐Li_2_S/CNT electrodes were used as a working electrode and lithium metal foil (half batteries) or graphite electrode (full batteries) (provided by Samsumg Company) were used as a counter electrode. Celgard 2400 and 3 m‐LiTFSI in DOL/DME (=85/15) were used as a separator and an electrolyte, respectively. All the batteries were assembled in an argon‐filled glove box (H_2_O and O_2_ content: <1 ppm). For charge/discharge behavior at a constant current density, the Li/Li_2_S batteries were first charge to 4.0 V then discharged to 1.5 V at a rate of 0.1 C (1 C = 1166 mA g^−1^). After that, the battery was cycled at a potential range from 1.5 to 3.0 V at a rate of 0.2 C. For the graphite/Li_2_S full batteries (the capacity ratio of graphite anode and Li_2_S cathode is around 1.05–1.1:1), the batteries were first charged to 3.8 V and then discharged to 1.0 V at a rate of 0.1 C. Subsequently, the batteries were cycled at a rate of 0.2 C/1 C with a potential range from 1.0 to 3.0 V. The rate capabilities of the graphite/holey‐Li_2_S battery were evaluated in a successive manner by varying the charge/discharge current density as 0.1, 0.2, 0.3, 0.4 0.5, 0.6, 0.8, and 1 C, and finally went back to 0.1 C, respectively.

## Conflict of Interest

The authors declare no conflict of interest.

## Supporting information

Supplementary
