# Supplementary material for: An Ultrahigh Capacity Graphite/Li2S Battery with Holey‐Li2S Nanoarchitectures
Source: Adv Sci (Weinh). 2018 May 7;5(7):1800139. doi: 10.1002/advs.201800139 (PMC6051237; doi:10.1002/advs.201800139)
Supplement: Supplementary file 1 — Supplementary [file ADVS-5-1800139-s001.pdf]

Copyright WILEY-VCH Verlag GmbH & Co. KGaA, 69469 Weinheim, Germany, 2018.

ADVANCED SCIENCE
Open Access

Supporting Information

for *Adv. Sci.,* DOI: 10.1002/advs.201800139

An Ultrahigh Capacity Graphite/Li_2S Battery with Holey-Li_2S Nanoarchitectures

*Fangmin Ye, Hyungjun Noh, Hongkyung Lee, and Hee-Tak Kim**

Copyright WILEY-VCH Verlag GmbH & Co. KGaA, 69469 Weinheim, Germany, 2018.

Supporting Information

An Ultrahigh Capacity Graphite/Li_2S Battery with Holey-Li_2S Nanoarchitectures

*Fangmin Ye, Hyungjun Noh, Hongkyung Lee, and Hee-Tak Kim**

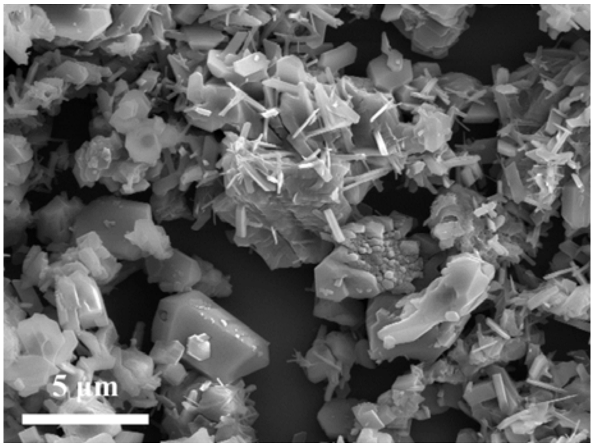

Figure S1. SEM image of the commercial Li_2SO_4•H_2O powder.

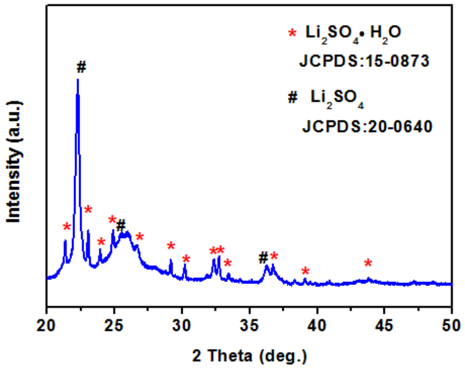

Figure S2. XRD pattern of the as-prepared plate-Li_2SO_4/CNT sample.

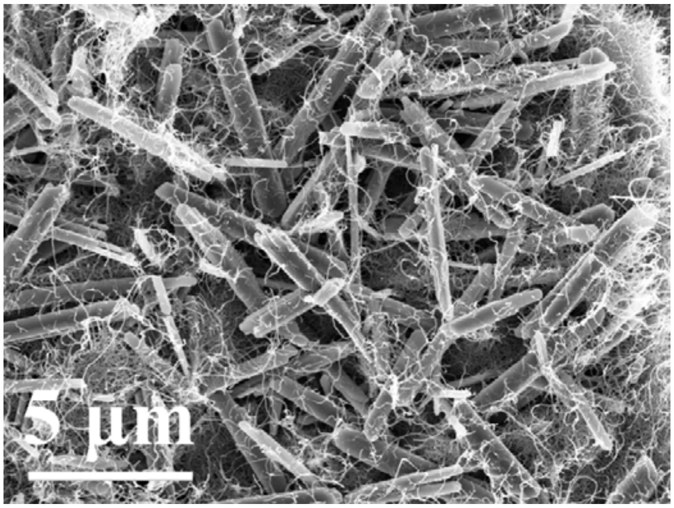

Figure S3. A SEM image of the Li_2SO_4/CNT composite prepared by using PVP as a surfactant.

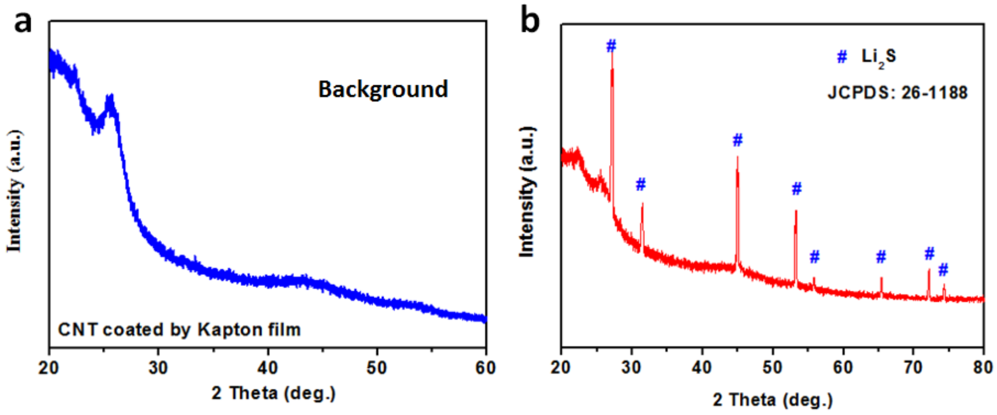

Figure S4. XRD pattern for the as-converted holey-Li_2S/CNT nanoarchitectures.

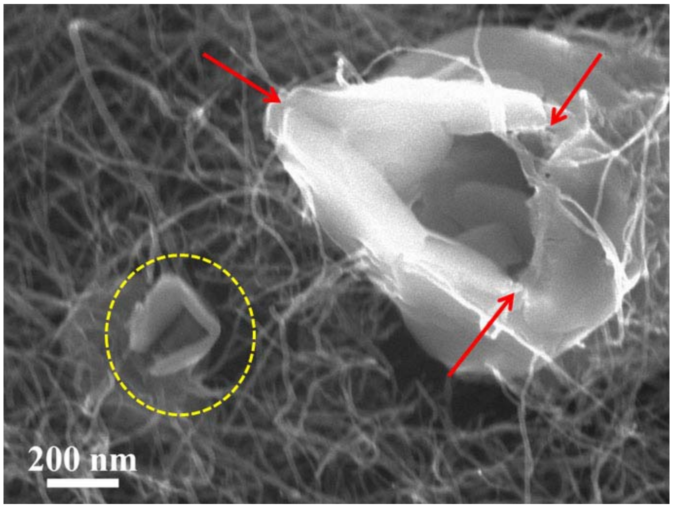

Figure S5. A SEM image supporting the self-assembly process of the holey-Li_2S structure.

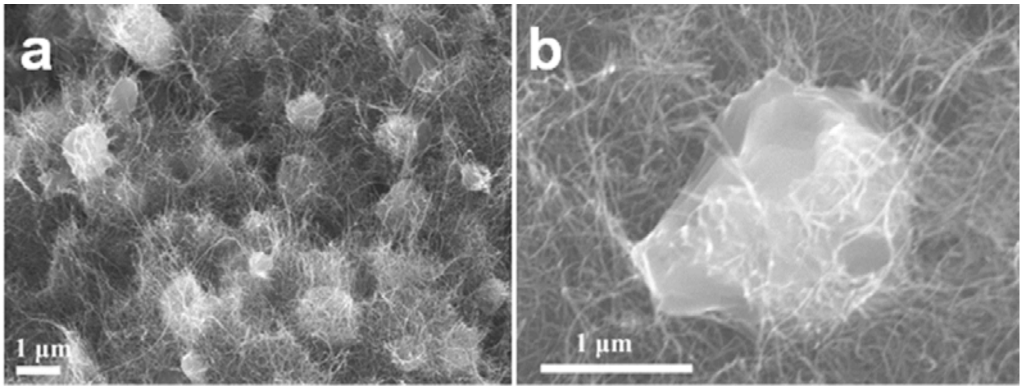

Figure S6. SEM images of the solid-Li_2S/CNT electrodes cathode after a further heat treatment of the holey-Li_2S/CNT electrodes at 1000 ^{o}C for 3 h.

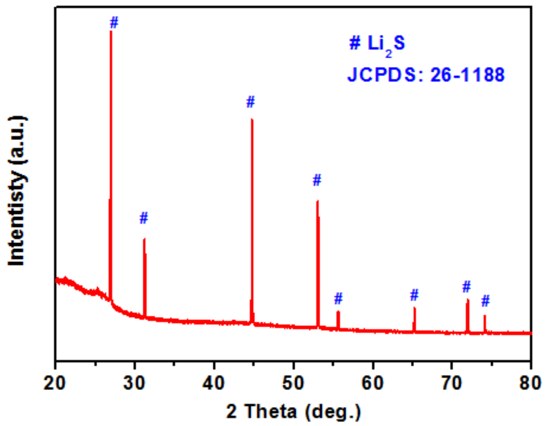

Figure S7. XRD pattern for the as-prepared solid-Li_2S/CNT cathode.

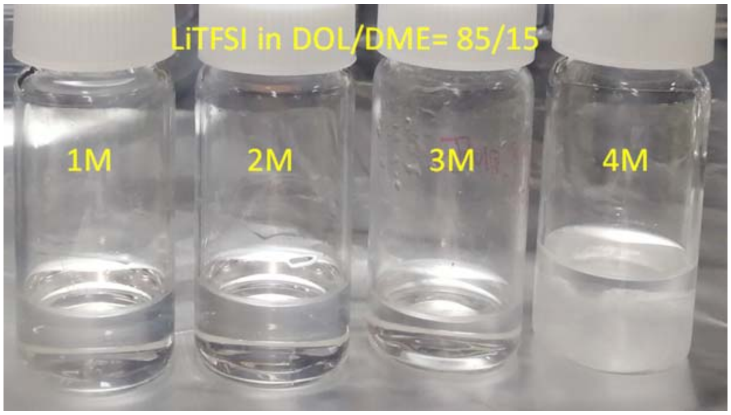

Figure S8. Photography of the 1, 2, 3 and 4M LiTFSI electrolytes in DOL/DME=85/15 in volume.

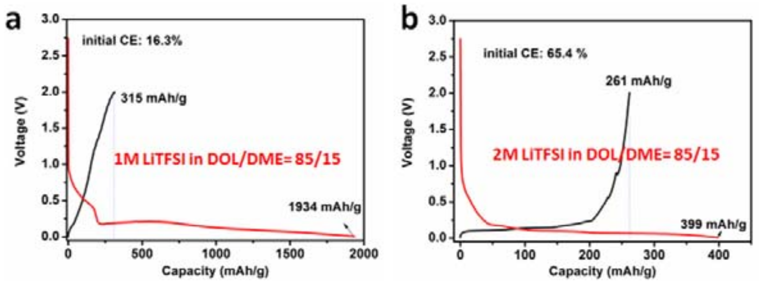

Figure S9. The initial discharge/charge curves of graphite/Li cell based on electrolytes with 1 and 2M LiTFSI in DOL/DME=85/15 in volume.

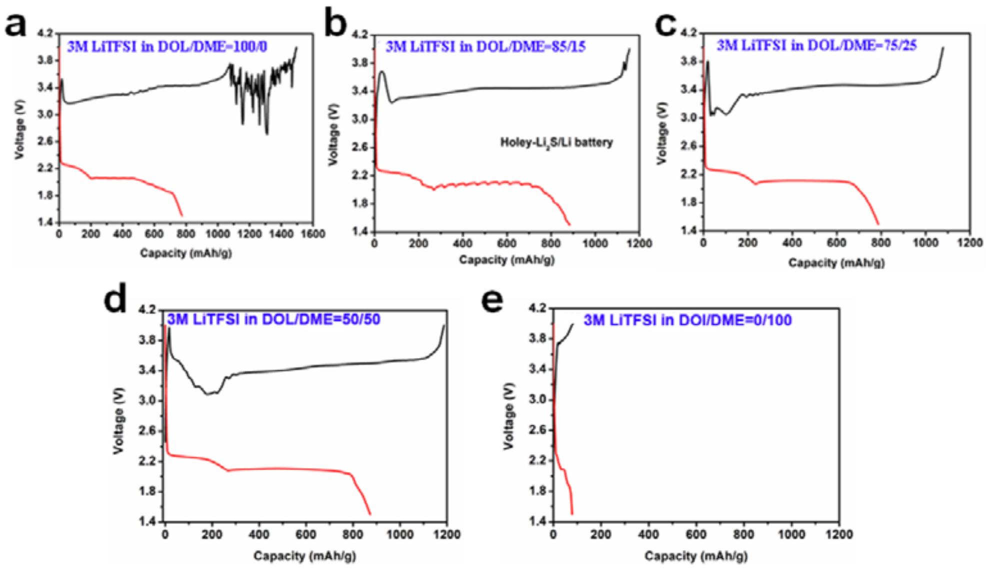

Figure S10. The first charge and discharge profiles of the Li_2S/Li half cells based on the DOL-rich electrolytes with different DOL/DME ratios.

Table S1. Comparison on the ion conductivity of various DOL-rich electrolytes

Electrolyte	Ion conductivity (mS/cm)
3M LiTFSI in DOL/DME=100/0	1.61
3M LiTFSI in DOL/DME=85/15	2.92
3M LiTFSI in DOL/DME=75/25	2.47
3M LiTFSI in DOL/DME=50/50	2.18
2M LiTFSI in DOL/DME=85/15	2.21
1M LiTFSI in DOL/DME=85/15	1.68

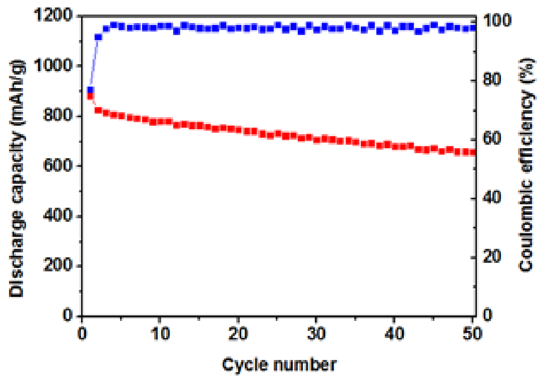

Figure S11. Cycling performance of the Li/holey-Li_2S battery at 0.2 C by using the 3 M LiTFSI DOL/DME=85/15 electrolyte.

Table S2. The comparison on the electrochemical performances of the reported graphite/Li_2S batteries.

Cathode/Anode	Li_2S Loading (mg/cm^2)	Test condition		Electrolyte	Cycling capacity (based on Li_2S)	Ref.
		activation and voltage cutoff	cycling voltage range			
Graphene-Li_2S/Graphite	2.2	C/12, 4.4 V	0.5-3.5 V	1M $[Li(G4)_{0.8}][TFSA]$/HFE	~ 410 mAh/g, **C/12** after 100 cycles	1
KB-Li_2S/Graphite	--	0.1 C, 3.0 V	0.1-3.0 V	TRIDME/LITFSI/HFE (molar ratio: 1:1:4)	~ 224 mAh/g, **0.1C** after 50 cycles	2
**Li_2S@Graphene/Graphite**	2	0.1 C, 3.5 V	1.4-3.0 V	1M D2/DOL (volume ratio: 2:1)	~ 308 mAh/g, **0.1C** after 200 cycles	3
Li_2S-MC/Graphite	<1	0.1 C, 3.0 V	1.0-3.0 V	1M $LiPF_6$ in EC/DEC	~600 mAh/g , **0.1C** after 150 cycles	4
Li_2S@PC/Graphite	1	0.1 C, 4.0 V	1.3-2.8 V	1M LiTFSI in DOL/DME With 1 wt% $LiNO_3$	~173 mAh/g, **0.5C** after 100 cycles	5
**holey-Li_2S/graphite**	**2.1**	**0.1 C, 3.8 V**	**1.0-3.0 V**	3M LiTFSI in DOL/15 v.% DME	**810 mAh/g, 0.1C for 1st discharge**<br>**583 mAh/g, 0.2C after 100 cycles**<br>**300 mAh/g, 1C after 600 cycles**	**Our work**

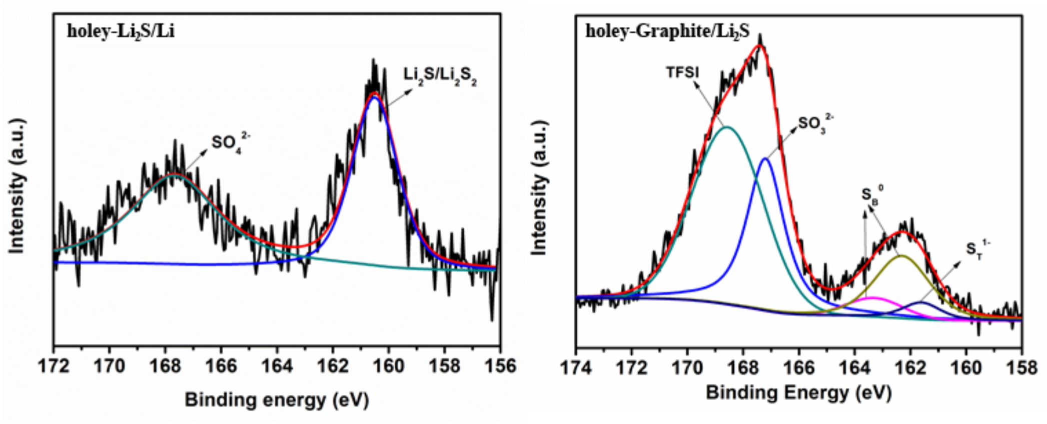

Figure S12. S_{2p} XPS results for the lithium metal and graphite electrode collected after the initial charge of the Li/holey-Li_2S and graphite/holey-Li_2S batteries, respectively.

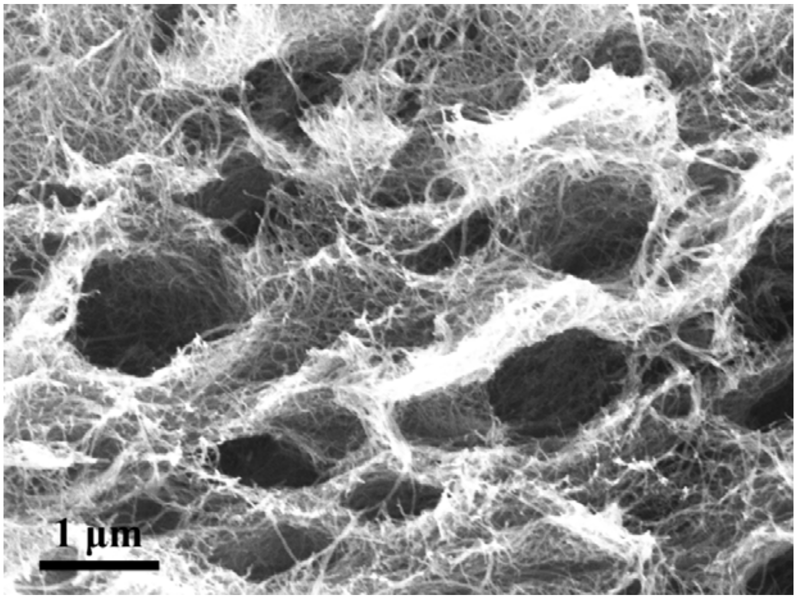

Figure S13. SEM image of the cathode after the initial charge of the graphite/holey-Li_2S battery.

Table S3. Several parameters of the graphite/$LiCoO_2$ and graphite/holey-Li_2S batteries.

	**Graphite/$LiCoO_2$**	**Graphite/holey-Li_2S**
**cathode (mg)**	13.31	5
**anode (mg)**	6.95	7.85
**electrolyte**	1M $LiPF_6$ in EC/DEC	3M LiTFSI in DOL/DME=85/15 (v/v)
**current density and average discharge potential**	56 mA/g; 3.70V	56 mA/g; 1.93V
**discharge capacity (mAh)**	1.13	1.80
**specific energy density (**based on the mass of cathode and anode**)**	56 mA/g; 206 Wh kg^{-1}	56 mA/g; 270 Wh kg^{-1}

The specific energy was calculated by using the following equation.

$$E = \frac{V_a \times C}{M} \quad (1)$$

Where, E is specific energy density of battery (Wh/kg); V_a is the average discharge potential (V), which can be determined by the integration on discharge curve; C is the discharge capacity of battery (Ah) and M is the total weight of anode and cathode electrode (kg).

References

1. Z. Li, S. Zhang, S. Terada, X. Ma, K. Ikeda, Y. Kamei, C. Zhang, K. Dokko, M. Watanabe, *ACS Appl. Mater. Interfaces* **2016**, 8, 16053.

2. Y. Wu, T. Yokoshima, H. Nara, T. Momma, T. Osaka, *J. Power Sources* **2017**, 342, 537.

3. G. Tan, R. Xu, Z. Xing, Y. Yuan, J. Lu, J. Wen, C. Liu, L. Ma, C. Zhan, Q. Liu, T. Wu, Z. Jian, R. Yassar, Y. Ren, D. J. Miller, L. A. Curtiss, X. Ji, K. Amine, *Nature Energy* **2017**, 2, 17090.

4. S. Zheng, Y. Chen, Y. Xu, F. Yi, Y. Zhu, Y. Liu, J. Yang, C. Wang, *ACS Nano* **2013**, 7, 10995.

5. N.Wang, N. Zhao, C. Shi, E, Liu, C. He, F. He, L. Ma, *Electrochimica Acta* **2017**, 256, 348.
